# Organic carbon budget for the eastern boundary of the North Atlantic subtropical gyre: major role of DOC in mesopelagic respiration

**DOI:** 10.1038/s41598-017-10974-y

**Published:** 2017-08-31

**Authors:** Yeray Santana-Falcón, Xosé Antón Álvarez-Salgado, María Dolores Pérez-Hernández, Alonso Hernández-Guerra, Evan Mason, Javier Arístegui

**Affiliations:** 1 Instituto de Oceanografía y Cambio Global, IOCAG, Universidad de Las Palmas de Gran Canaria, ULPGC, 35017 Las Palmas de Gran Canaria, Spain; 2 CSIC, Instituto de Investigaciones Marinas, Eduardo Cabello, 6, 36208 Vigo, Spain; 3 Department of Physical Oceanography, Woods Hole Oceanographic Institution, Woods Hole, Massachusetts, USA; 4 Instituto Mediterráneo de Estudios Avanzados, CSIC-UIB. C. Miquel Marquès, 21, 07190 Esporles, Illes Balears, Spain

## Abstract

Transports of suspended particulate (POC_susp_) and dissolved (DOC) organic carbon are inferred from a box-model covering the eastern boundary of the North Atlantic subtropical gyre. Corresponding net respiration rates (R) are obtained from a net organic carbon budget that is based on the transport estimates, and includes both vertical and lateral fluxes. The overall R in the mesopelagic layer (100–1500 m) is 1.6 ± 0.4 mmol C m^−2^ d^−1^. DOC accounts for up to 53% of R as a result of drawdown of organic carbon within Eastern North Atlantic Central Water (ENACW) that is entrained into sinking Mediterranean Overflow Water (MOW) that leads to formation of Mediterranean water (MW) at intermediate depths (~900 m). DOC represents 90% of the respired non-sinking organic carbon. When converted into oxygen units, the computed net respiration rate represents less than half the oxygen utilization rates (OUR) reported for the mesopelagic waters of the subtropical North Atlantic. Mesoscale processes in the area, not quantified with our approach, could account in part for the OUR differences observed between our carbon budget and other published studies from the North Atlantic, although seasonal or interannual variability could also be responsible for the difference in the estimates.

## Introduction

Oceanic respiration occurs over the entire water column^1, 2^, as opposed to photosynthesis, which is restricted to the epipelagic layer (roughly above 150 m). The non-respired fraction of the biogenic organic carbon produced in the epipelagic layer is susceptible to downward export in the form of dissolved (DOC) and particulate (POC) organic carbon, supporting respiration in the dark ocean^2, 3^. At the global scale, the relative contribution of DOC to oxygen consumption in the dark ocean has been estimated to be 10–20%^4, 5^, but it may increase significantly in regions of deep-water convection^5–7^. Paradoxically, the sum of DOC and sinking POC collected with sediment traps does not frequently account for the estimated dark ocean respiration rates^8–10^. This conflicting imbalance could be a consequence of methodological uncertainties in the estimation of respiration rates and/or carbon fluxes^10–12^. For instance, the bulk of particulate material in the water column is composed of slow-settling or suspended particles^13–15^ that can escape from sediment traps due to their buoyancy^16^. Several studies have suggested that suspended particles are an important source of organic matter for prokaryotic organisms in the mesopelagic zone^17, 18^. Therefore, suspended particles would represent an unquantified source of “missing” organic carbon, especially in areas where lateral advection is intense, such as in Eastern Boundary Current systems^19^. Indeed, it has been already shown that, using a box-model approach, suspended particulate organic carbon (POC_susp_) could support up to 59% of the total mesopelagic respiration in the southwestern sector of the Canary Current^20^, a region strongly affected by the coastal-ocean export of particulate material from the NW African coastal upwelling system^21, 22^.

**Figure 1:**
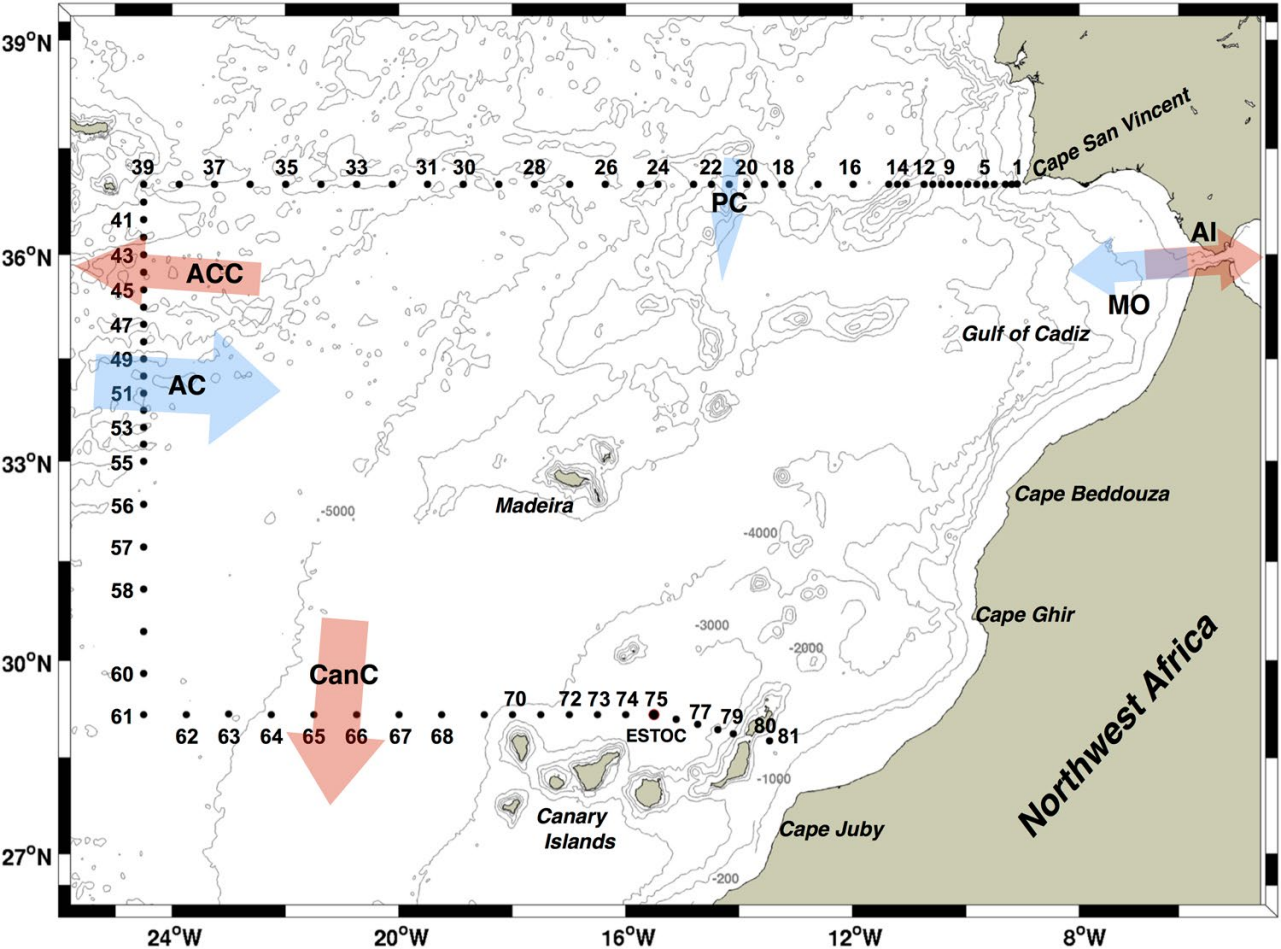
Location of the CTD stations. Biogeochemical stations are numbered. AC, ACC, CanC, and PC refer to Azores Current, Azores Counter-Current, Canary Current, and Portugal Current. AI and MO indicate the surface Atlantic inflow and bottom Mediterranean overflow. Blue/red arrows indicate transport into/out of the study region. Station 75 is at the ESTOC site. The map was produced using M_Map toolbox (https://www.eoas.ubc.ca/~rich/map.html) on MATLAB R2013a, https://www.mathworks.com/products/matlab/. Global ETOPO is used for bathymetric data (https://www.ngdc.noaa.gov/mgg/global/).

In the present study, following the approach used by Alonso-González and coworkers in the southern Canary Current^20^, we estimate the contribution of DOC and POC_susp_ fluxes to net respiration in the eastern boundary of the North Atlantic subtropical gyre (Fig. 1). Our overarching hypothesis is that, contrary to the southern Canary Current where POC_susp_ is an important component of the carbon budget^20, 23^, the DOC contribution to total respiration in the study area is larger than that of POC_susp_. The two areas are oceanographically distinct. On the one hand, the coastline orientation in our region is unfavorable to coastal upwelling, which is absent or limited just to the summer months^24, 25^. On the other hand, our region is characterized by the density-driven exchange of water masses between the Mediterranean Sea and the eastern North Atlantic^26^. As a consequence, Eastern North Atlantic Central Water (ENACW) is entrained into the dense fast-flowing Mediterranean Overflow Water (MOW) that exits through the Strait of Gibraltar^27–29^ and is subducted along the slope into the Gulf of Cádiz (ca. 33–37°N)^30–33^ to below 1000 m depth, where it spreads into the Atlantic as Mediterranean Water (MW)^27–29, 34–37^. This entrainment of ENACW into MOW may promote the downward flux of DOC to intermediate layers in this particular region, as has already been shown for anthropogenic carbon dioxide^38^.

In a previous study^39^, based on a rough estimation of organic carbon fluxes in a box region that included the Strait of Gibraltar, it has been suggested that DOC remineralization could explain about 90% of the inorganic carbon produced in the water column. Our study tests this hypothesis, providing direct measurements of POC_susp_ and DOC fluxes for integration within a box-model analysis. We aim to contribute to the understanding of the organic carbon dynamics in this complex region, which is characterized by the confluence of water masses of different origin, water mass entrainment and subduction, and intermittent upwelling^24, 40–43^.

## Results

### Organic carbon distributions

**Figure 2:**
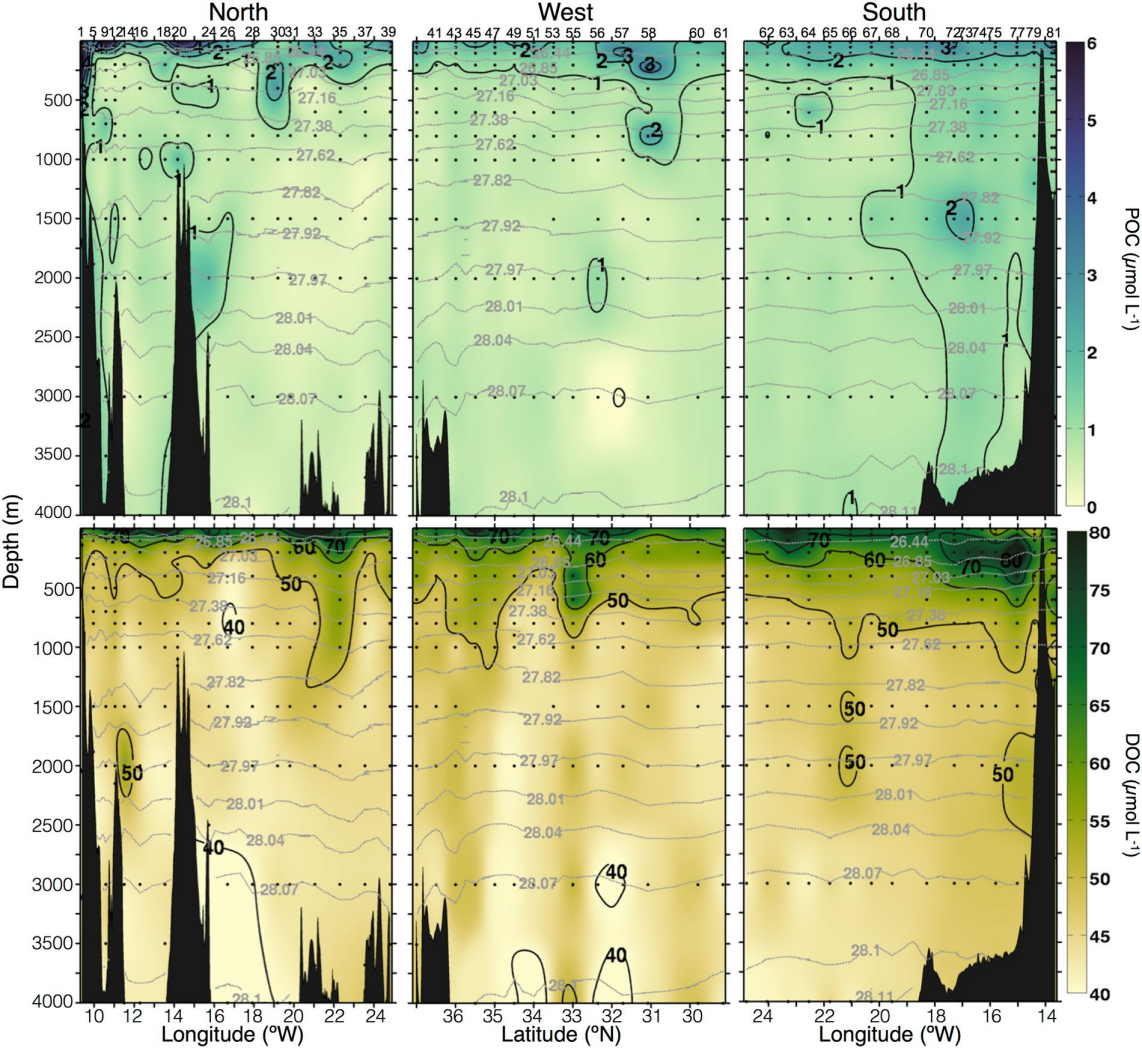
Vertical section (0–4000 m) of POC_susp_ (µmol C L^−1^; upper panels) and DOC (µmol C L^−1^; lower panels) for the northern (left), western (middle), and southern (right) transects. Biogeochemical stations are indicated at the top of the upper panels. Gray dotted lines indicate neutral density.

The DOC and POC_susp_ data used in this work were collected during a research cruise in autumn 2009. The cruise consisted of a set of hydrographic stations distributed regularly along three transects (northern, western and southern) that delimit the box presented in Fig. 1 (see Materials and Methods for further details on sampling and analysis). POC_susp_ and DOC distributions from 0 to 4000 m (Fig. 2) show that surface POC_susp_ concentrations along the northern transect decrease offshore from 4–6 µmol C L^−1^ in the coastal area to about 2 µmol C L^−1^ in the open ocean. Below 500 m POC_susp_ concentrations are 1 µmol C L^−1^ or less, except around a seamount at ~14° W. Waters with relatively high POC_susp_ deepen at station 30. Surface DOC concentrations are higher than 60 µmol C L^−1^ for the entire transect. A deepening of DOC occurs at stations 30–37.

Surface POC_susp_ concentrations along the western transect are higher than 1–2 µmol C L^−1^. A deepening of POC_susp_-rich waters at stations 56–58 coincides with the shoaling of isoneutral surfaces between two mesoscale eddies^44^. POC_susp_ concentrations remain around 1 µmol C L^−1^ along the rest of the section with little variation. DOC is about 60–70 µmol C L^−1^ near the surface, and decreases to less than 50 µmol C L^−1^ with depth. Central waters with high DOC concentrations descend along sloping isoneutrals at stations 53–56. The deepening of DOC north of 35°N coincides with the location of the Azores Current system^44^.

The southern transect shows surface POC_susp_ concentrations of 2–3 µmol C L^−1^. A westward decrease is observed below 250 m. Surface DOC concentrations show values above 70–80 µmol C L^−1^. A patch of relatively high POC_susp_ below 1000 m depth coincides with cool and fresh waters^44^. DOC shows values above 70–80 µmol C L^−1^ at the surface, with maximum concentrations above 100 µmol C L^−1^ at subsurface depths (~200 m) in the eastern stations close to the Lanzarote Passage. Intermediate waters (below the 27.38 isoneutral) show low DOC concentrations, except at stations 64–65 where a front between a cyclonic eddy and the southward Canary Current is periodically observed^44^.

### Mass transports

**Figure 3:**
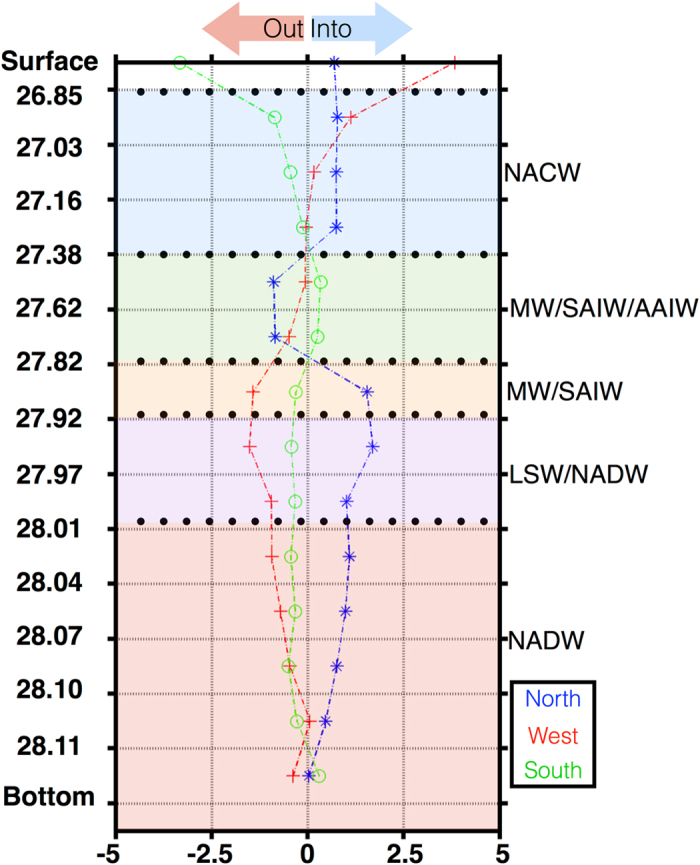
Integrated mass transports for the northern (blue diamonds over a dashed line), western (red crosses over a dotted line) and southern (green circle dashed line) transects. The sign of the net transport is negative/positive for diverging/converging flow out of/into the box. North Atlantic Central Water (NACW), Mediterranean Water (MW), sub-Artic Intermediate Water (SAIW), Antarctic Intermediate Water (AAIW), Labrador Sea Water (LSW), and North Atlantic Deep Water (NADW) water masses are indicated both by color and acronym. Y-axis is neutral density.

Mass transports across the northern, western, and southern transects are taken from a previous study by Pérez-Hernandez and co-workers who developed an inverse box model with data from the same cruise^44^ (see Material and Methods for further details about the inverse model and its reliability). A summary of the integrated lateral mass transport at each transect is presented in Fig. 3. Note that fluxes into/out of the box are indicated by positive/negative signs, respectively.

A predominant inward (southward) transport is observed across the northern transect (~37°N; blue line), interrupted only by the outward (northward) transport of intermediate waters (isoneutrals 27.38 to 27.92). The mass transport across the western transect (~24°W; red line) is strongly inwards (eastward) in the surface and central waters, and predominantly outwards (westward) below. The mass transport across the southern transect (~29°N; green line) is outwards (southward) in the upper 700 meters of the water column, while the flow of intermediate and deep waters varies by less than ±2 Sv.

### POC_susp_ and DOC transports

Organic carbon transports along isoneutrals are calculated by multiplying the corresponding POC_susp_ and DOC concentrations (Fig. 2) by the mass transports (Fig. 3) (see Materials and Methods for more details on the estimation and robustness of the carbon transports). For the case of DOC, transports of the non-refractory pool DOC (e-DOC) are calculated. The e-DOC pool is obtained by subtracting a background refractory DOC concentration (~45 mmol C m^−^³ in the study area) from the measured DOC. We only consider the e-DOC fraction because the refractory fraction renewal is thousands of years^45, 46^, a period much longer than the decadal renewal time for the study box.

**Figure 4:**
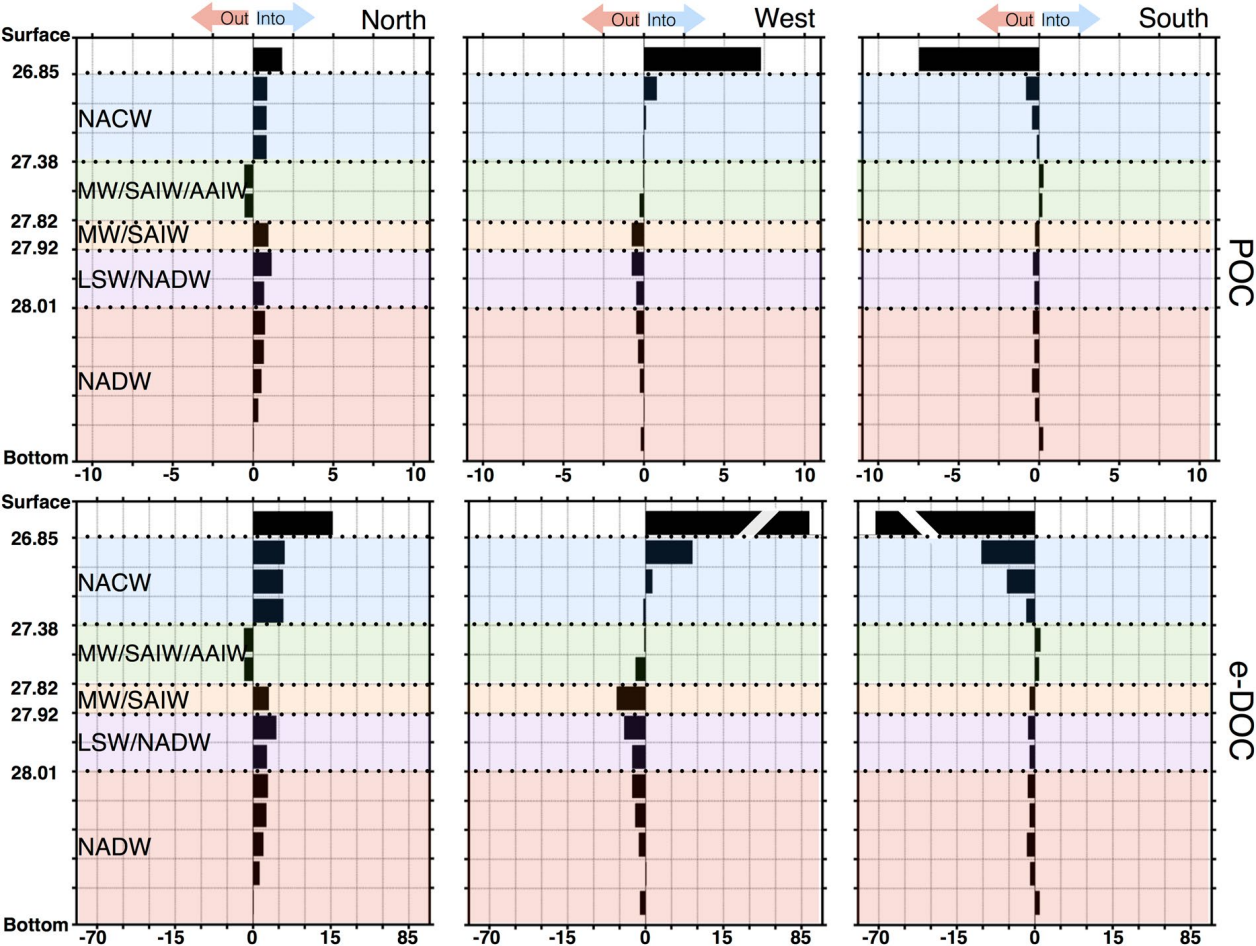
POC_susp_ (upper panels) and DOC (lower panels) fluxes (10^8^ mol C d^−^¹) at each transect. Positive/negative values indicate inputs/outputs to/from the box. Water masses are indicated both by color and acronym: North Atlantic Central Water (NACW), Mediterranean Water (MW), sub-Arctic Intermediate Water (SAIW), Antarctic Intermediate Water (AAIW), Labrador Sea Water (LSW), and North Atlantic Deep Water (NADW). Y-axes is neutral density.

POC_susp_ and e-DOC transports in the area (Fig. 4) may be divided into a southward flux of surface and central waters, interrupted by a weak northward transport of the upper intermediate waters, that is observed both at the northern and southern transects. Southward transport prevails again in the deepest waters. The zonal circulation is predominantly eastward at the surface and central waters. The trend reverses in the intermediate and deep layers where a westward transport is observed, especially at the bottom of the intermediate layer.

POC_susp_ and e-DOC fluxes in the surface, central, intermediate, and deep waters at each transect are summarised in Table 1. The inputs (positive values) of organic carbon through the northern and western transects exceed the outputs (negative values) of the southern transect at both surface and central layers. Weak horizontal output fluxes are found both in the intermediate and the deepest layers.

**Table 1:** POC_suspand_ e-DOC fluxes (10^8^ mol C d^−1^) for surface, central, intermediate, and deep waters for the northern, western, and southern transects. Error bars are given. Positive/negative values indicate input/outputs to/from the box.

	Northern Transect		Western Transect		Southern Transect		Global	
	POC_susp_	e-DOC	POC_susp_	e-DOC	POC_susp_	e-DOC	POC_susp_	e-DOC
*Surface*	1.79 ± 0.02	15.29 ± 0.04	7.27 ± 0.2	85.20 ± 2.8	−7.45 ± 0.5	−70.46 ± 3.1	**1.48** ± **0.2**	**28.51** ± **2.0**
*Central*	2.52 ± 0.3	17.83 ± 2.3	0.87 ± 0.03	9.97 ± 0.7	−1.34 ± 0.1	−17.46 ± 2.9	**1.93** ± **0.2**	**−0.12 ± 0.06**
*Intermediate*	−0.12 ± 0.06	−0.39 ± 0.2	−1.03 ± 0.09	−7.73 ± 0.9	0.22 ± 0.01	0.88 ± 0.02	**−0.96** ± **0.1**	**−7.53** ± **0.5**
*Deep*	4.04 ± 0.4	16.04 ± 1.5	−2.41 ± 0.3	−13.24 ± 0.9	−1.68 ± 0.1	−5.95 ± 0.5	**−0.24** ± **0.3**	**−3.79** ± **1.0**

## Discussion

**Figure 5:**
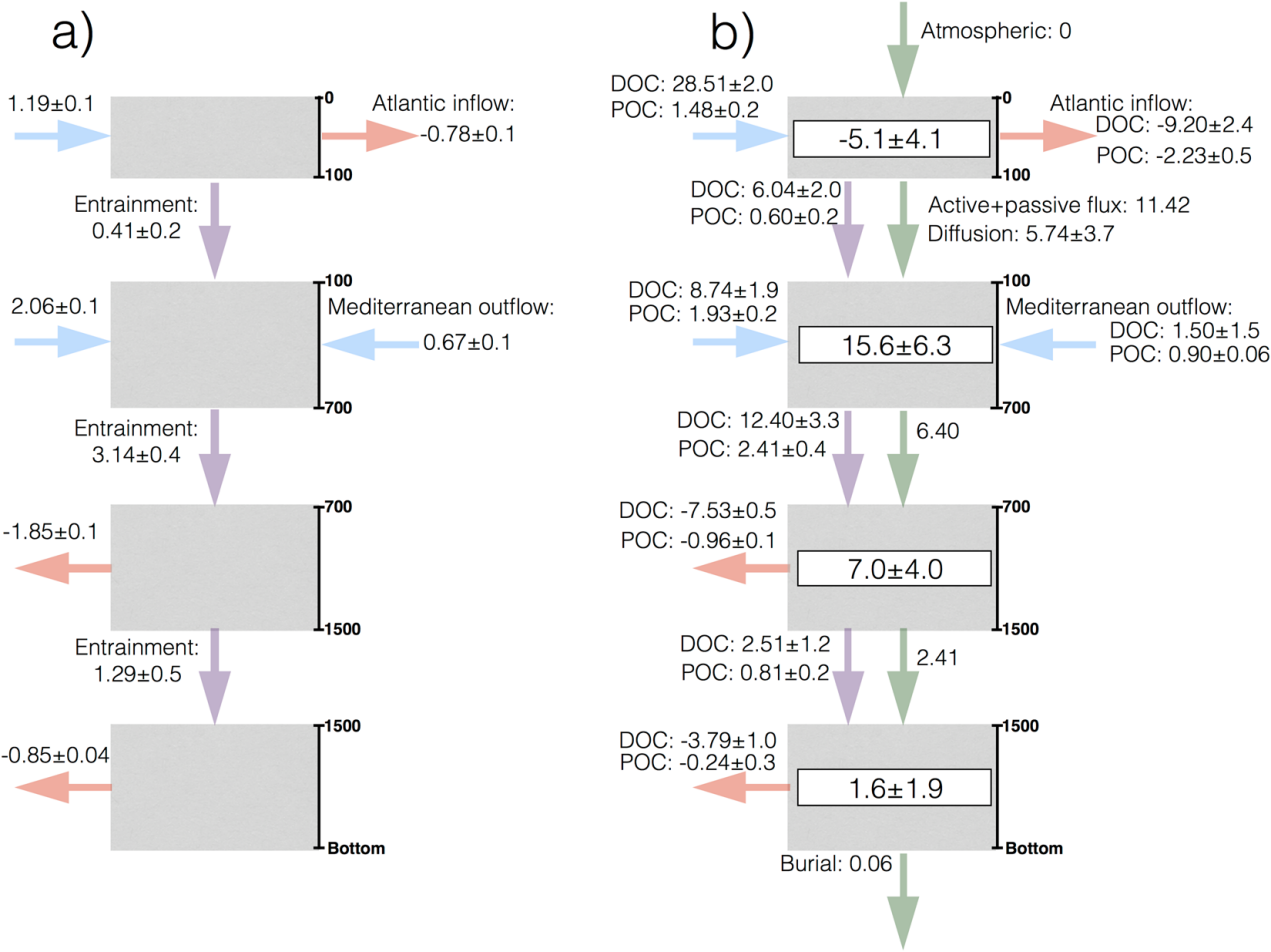
Schematic diagram showing (**a**) the mass transport and (**b**) the organic carbon budgets in the box region. The water column is divided into surface (0–100 m), central (100–700 m), intermediate (700–1500 m), and deep (below 1500 m) layers. Arrows indicate net transport (Sv) or organic carbon (10^8^ mol C d^−^¹) across the open boundaries. Light blue indicates lateral inputs (positive values), light red indicates lateral outputs (negative values), light violet indicates vertical entrainment, light green indicates vertical fluxes from the layer above. Net respiration rates for each compartment are indicated.

To evaluate the organic carbon sources within the box we build a mass balance (Fig. 5a) and an associated organic carbon budget (Fig. 5b). For the surface waters (0–100 m), 1.2 ± 0.1 Sv enter the box through the northern, southern and western transects, and −0.78 ± 0.1 Sv leave the box as the Atlantic inflow into the Mediterranean Sea (see Materials and Methods). As a result, the mass balance of inputs minus outputs to the box would be 0.4 ± 0.2 Sv. We propose that this volume is transported from the surface to the central waters to partially balance the loss of ENACW to intermediate waters when entrained into the MOW (see below the discussion about the mass balance of the central waters). The balance of organic carbon, i.e. POC_susp_ and e-DOC, transported across the northern, western and southern transects indicates that the surface waters of the box receive an external input from the Atlantic Ocean of 30.0 ± 2.0 × 10^8^ mol C d^−^¹. At the same time, according to our estimates (see Materials and Methods), −11.4 ± 2.9 × 10^8^ mol C d^−^¹ is exported within surface waters to the Mediterranean Sea. This number lies within the range of the Atlantic inflow of TOC previously measured in April 1998 (7.6 × 10^8^ mol C d^−^¹)^47^, and in September 1997 (27.1 × 10^8^ mol C d^−^¹)^48^, after subtraction of refractory DOC.

Furthermore, the non-respired organic carbon in surface waters may be transported to the dark ocean by turbulent diffusion, passive sinking, and active transport^49, 50^. We estimated first the vertical turbulent diffusion at 100 m depth as F = −ε × R/(N^2^ × (1 − R)) × (DOC_z2_ − DOC_z1_)/(z_2_ − z_1_)^51^. We considered (1) the averaged DOC in the upper 100 m (DOC_z1_ centered at z_1_ = 50 m) and from 100 to 200 m depth (DOC_z2_, centered at z_2_ = 150 m) for the calculation of the vertical DOC gradient, (2) the square of the Brunt-Väisälä frequency (N^2^) between 50 and 150 m, and (3) constant values for the dissipation rate (ε = 10^−8^ m^3^ s^−2^) and the Richardson number (R = 0.2). We obtained an averaged value of 0.4 ± 0.3 mmol C m^−^² d^−^¹, (average ± SD of 47 stns) that would give a total downward DOC flux of about 5.7 ± 3.7 × 10^8^ mol C d^−^¹, when multiplied by the surface area of the box (1.4 × 10^6^ km^2^).

Sinking POC fluxes obtained with surface tethered traps at 200 m depth and extrapolated to 150 m with the Martin equation^52^ were approximately 0.55 mmol C m^−^² d^−^¹ at the European Station for Time-series in the Ocean (ESTOC)^53^. In the northeast Atlantic (39–43°N, 17–19°W), the minimum vertical POC fluxes were estimated to be 0.36 ± 0.09 mmol C m^−^² d^−^¹ from drifting traps at 200 m depth during late summer^54^. Although the use of drifting traps has been frequently criticized for underestimating export fluxes^55–57^, comparison with POC fluxes at the Bermuda Time Series Station (BATS) derived from both surface-tethered traps and Thorium-234^56^ shows reasonable agreement during low productivity periods. At the ESTOC site, the lowest productivity values have been obtained during autumn (~10 mmol C m^−^² d^−^¹)^57^; considering an e-ratio (export/production ratio) of 5–10%^55^, the vertical POC flux during the less productive season would be 0.5–1.0 mmol C m^−^² d^−^¹. Therefore, an average value of the export rate from drifting traps of 0.36 to 1.0 mmol C m^−^² d^−^¹ is representative of the sinking flux from surface waters within the box at that time of the year. Aggregation and disaggregation of sinking particles by biotic and abiotic mechanisms could modify the sinking rates and magnitude of POC fluxes with depth across the dark ocean^58–62^, although our box model approach does not allow identification of these processes.

In addition, the estimated averaged vertical flux of POC mediated by migrant zooplankton (active flux) was 0.22 mmol C m^−^² d^−^¹ south of the island of Gran Canaria^63^, accounting for 25% of the sinking POC flux (passive flux) in the region. More recently, a mean active flux of 0.28 mmol C m^−^² d^−^¹ (29% of the passive flux) has been estimated north of Gran Canaria^64^; these estimates agree with those observed at BATS (0.17 mmol C m^−^² d^−^¹)^65^ and the Hawaiian Ocean Time Series (HOT; 0.26 mmol C m^−^² d^−^¹)^66^. However, the active flux accounted for only 8% and 11–44% of the passive flux at their respective regions^65, 66^. If we assume that the active flux represents at best 20% of the passive flux in our region, the passive + migratory POC flux from the surface to the central waters would be around 0.82 mmol C m^−^² d^−^¹ which, multiplied by the area of our box, yields a total POC flux from the surface to the central waters of 11.4 × 10^8^ mol C d^−^¹.

The central waters (100–700 m) within the box receive an overall lateral input of POC_susp_ and e-DOC of 10.7 ± 1.9 × 10^8^ mol C d^−^¹ from the surrounding Atlantic Ocean and 2.4 ± 1.5 × 10^8^ mol C d^−^¹ from the Mediterranean outflow through the Strait of Gibraltar. In addition, it is well known that MOW is subducted within the study box to more than 1000 m and that the central waters are entrained into the MOW as it sinks^29, 38^. In fact, the mass balance of central waters within the box (Fig. 5a), considering the net water flows across the northern, southern, and western transects (2.1 ± 0.1 Sv), the Mediterranean outflow into the Atlantic (0.67 ± 0.1 Sv; see Materials and Methods), and the input of 0.4 ± 0.2 Sv from the surface waters, is 3.1 ± 0.4 Sv. Therefore, in order to maintain conservation of volume in the central waters, 3.1 ± 0.4 Sv should enter the intermediate layer by entrainment of the central waters into the subducting Mediterranean outflow. Our volume calculation is in agreement with earlier estimates (2–3 Sv^67^; 2.6 Sv^68^; 1.3 Sv^28^; 2.3 Sv^38^). To obtain the organic carbon transported by this volume, we multiply the average organic carbon concentration at the bottom of the central layer by 3.1 ± 0.4 Sv to obtain a downward POC + e-DOC flux of 14.8 ± 3.7 × 10^8^ mol C d^−^¹, 90% of which is DOC. Similarly, we also calculate the organic carbon transport associated with the 0.4 ± 0.2 Sv of surface waters that enter the central waters yielding 6.6 ± 2.2 × 10^8^ mol C d^−^¹, 10% as POC_susp_ and 90% as DOC (Fig. 5b).

For the intermediate waters (700–1500 m), aside from the organic carbon that enters this layer by entrainment of ENACW into the subducting MOW (14.8 ± 3.7 × 10^8^ mol C d^−^¹), the other organic carbon transports associated with the water mass fluxes leave this layer. The net balance of POC_susp_ and e-DOC transport across the northern, western and southern transects is −8.5 ± 0.6 × 10^8^ mol C d^−^¹. This indicates an export from the box to the adjacent Atlantic Ocean, and is especially intense across the western transect (see Table 1), contrary to the surface and central waters where organic carbon was imported from the Atlantic Ocean. Furthermore, the mass balance of the intermediate waters (Fig. 5a) indicates that there should be an entrainment of 1.3 ± 0.5 Sv into the deep waters for volume conservation, that translates into a drawdown of organic carbon of 3.3 ± 1.4 × 10^8^ mol C d^−^¹, 76% of which is DOC (Fig. 5b). In addition, an average particle flux below 700 m of 0.46 mmol C m^−^² d^−^¹ has been obtained from three sites located within the Canary Current region^69^. Assuming this value for our region of study, we obtain a vertical POC supply to intermediate waters of 6.4 × 10^8^ mol C d^−^¹.

Finally, the mass transport balance of the deep waters (below 1500 m), 0.4 ± 0.4 Sv, is compatible with mass conservation within the uncertainty of the estimates. POC_susp_ + e-DOC transported from the intermediate waters by entrainment (3.3 ± 1.4 × 10^8^ mol C d^−1^), sinking POC (2.4 × 10^8^ mol C d^−1^), organic carbon burial into the sediments (0.06 × 10^8^ mol C d^−^¹)^70^, and the carbon balance at the open boundaries of the box (−4.0 ± 1.3 × 10^8^ mol C d^−^¹) yield a negligible carbon excess of 1.6 ± 1.9 × 10^8^ mol C d^−^¹. The sinking POC flux at 1500 m was derived from the flux at 700 m using the Martin equation^52^.

Overall, the organic carbon balance for the surface waters associated with the lateral fluxes across the northern, western and southern transects (30.0 ± 2.2 × 10^8^ mol C d^−^¹), losses from the Strait of Gibraltar (−11.4 ± 2.9 × 10^8^ mol C d^−^¹), entrainment (−6.6 ± 2.0 × 10^8^ mol C d^−^¹), active + passive transport fluxes (−11.4 × 10^8^ mol C d^−^¹), and turbulent diffusion (−5.7 ± 3.7 × 10^8^ mol C d^−^¹), yields −5.1 ± 4.1 × 10^8^ mol C d^−^¹. Assuming steady state conditions, this balance results in a net heterotrophy (R > 0) of about −0.4 ± 0.3 mmol C m^−^² d^−^¹ at the time of this study.

The overall balance of organic carbon for the central waters includes the POC_susp_ + DOC lateral fluxes across the Atlantic boundaries of the box (10.7 ± 2.1 × 10^8^ mol C d^−^¹; 82% as DOC) and the Strait of Gibraltar (2.4 ± 1.6 × 10^8^ mol C d^−^¹; 62% DOC), passive + active fluxes of POC from surface waters (11.4 × 10^8^ mol C d^−^¹), and to intermediate waters (−6.4 × 10^8^ mol C d^−^¹), downward DOC fluxes by turbulent diffusion (5.7 ± 3.7 × 10^8^ mol C d^−^¹), and entrainment from the surface (6.6 ± 2.2 × 10^8^ mol C d^−^¹), and to the intermediate waters (−14.8 ± 3.7 × 10^8^ mol C d^−^¹). All these organic carbon fluxes result in a net R of 15.6 ± 6.3 × 10^8^ mol C d^−^¹ (1.1 ± 0.4 mmol C m^−^² d^−^¹).

Lastly, for intermediate waters the POC_susp_ + e-DOC includes the balance across the northern, western and southern boundaries (−8.5 ± 0.6 × 10^8^ mol C d^−^¹), entrainment from central waters (14.8 ± 3.7 × 10^8^ mol C d^−^¹) and into deeper waters (−3.3 ± 1.4 × 10^8^ mol C d^−^¹), and vertical downward POC fluxes from central waters (6.40 × 10^8^ mol C d^−^¹) and to deeper layers (−2.4 × 10^8^ mol C d^−^¹), resulting in a net R of 7.0 ± 4.0 × 10^8^ mol C d^−^¹ (0.5 ± 0.3 mmol C m^−^² d^−^¹) assuming steady-state conditions.

The net R within the mesopelagic layer, assumed to be equivalent to the sum of the central and the intermediate waters, is 22.7 ± 5.2 × 10^8^ mol C d^−^¹ (1.6 ± 0.4 mmol C m^−^² d^−^¹). This value is lower than those obtained with a similar box model west of the Canary Islands^20^: 2.4–5.1 mmol C m^−^² d^−^¹. According to these authors, POC_susp_ would support 28–59% of the total mesopelagic R in that region. However, their estimates were based solely on POC_susp_ fluxes, assuming two scenarios in which the contribution of DOC was fixed at 15 and 30%. In the present study, we have measured DOC and calculated that it contributes about 53% to total mesopelagic R within the box. It has been hypothesized that DOC represents about 90% of the respiration of non-sinking organic carbon in the mesopelagic layer from an empirical carbon mass balance within a box bounded by the Strait of Gibraltar, 24–41° N and 22° W^39^. Our results indicate that DOC represents that fraction when only the non-sinking organic carbon is considered, in agreement with the high DOC consumption rates obtained in areas undergoing water mass transformation^6, 71^.

The estimated mesopelagic R is much lower than the oxygen utilization rates (OUR) calculated for the North Atlantic subtropical gyre^72–74^ (9–11 mmol C m^−^² d^−^¹ as converted using a -O_2_/C molar ratio of 1.4^75^). In addition, a carbon budget north of our box region during summer 2009^76^ estimated a total input of organic carbon to the twilight zone (ca. 50–1000 m) of ~7.7 mmol C m^−^² d^−^¹, due mainly to sinking POC. OUR values ranging from 4.7 to 16.6 mmol C m^−^² d^−^¹ between 100 to 750 m depth were obtained using tritium and ^228^Ra methodologies in the North Atlantic subtropical gyre^77^. Similar integrated values (4.9 to 13.0 mmol C m^−^² d^−^¹) of carbon respiration for the 100–1000 depth zone were obtained both by ^7^Be and POC flux attenuation methodologies in the subtropical North Atlantic^78^. A compilation of data from the Pacific ocean^79^ shows OUR values ranging from 0.04 to 18.1 mmol C m^−^² d^−^¹. In the North Pacific, OUR values integrated over the first 600 m depth^80^ averaged 4.6 mmol C m^−^² d^−^¹, while higher OUR values of 9.8 to 11.7 mmol C m^−^² d^−^¹ have been recently reported for a monthly time-series at HOT^12^. Values obtained during the present study might, however, underestimate the annual mean since our study took place during the unproductive autumn when POC_susp_ concentrations are particularly low^56^. Additionally, the region within the box is strongly influenced by mesoscale features such as filaments and eddies, whose effects on carbon sequestration remain to be quantified. The enhancement of carbon fluxes to the ocean interior driven by mesoscale eddies has been reported in a number of studies^81–88^. Indeed, injection of organic carbon from surface waters was especially intense in several areas, such as in the anticyclonic eddy at the northwestern corner of the box (stations 33–35), or the Azores Front (stations 52–55^44^). Moreover, upwelling filaments may play a key role in the coastal-ocean transport of organic matter^89, 90^. The Cape Ghir filament lies within the region of study, and may export to the open ocean 29 to 63% of the annual averaged primary production associated with the coastal upwelling^91, 92^. All of these features occur episodically within the box, and could enhance the supply of organic carbon to mesopelagic waters.

Near the ESTOC site, net R rates at the mesopelagic zone (ca. 150–700 m) of 2.0–3.1 mol C m^−^² y^−^¹ (5.5–8.5 mmol C m^−^² d^−^¹) were obtained by using a tracer conservation model applied to climatological data^93^. They also obtain consistent R rates based on ETS (electron transport system) respiratory activity for the same region at the end of March. Differences between these estimates and our values could be due to higher lateral advection of DOC and POC_susp_ from the coastal upwelling region at that site^56^, and higher productivity at the end of the late winter bloom.

In summary, this study provides an estimate of net respiration in the mesopelagic waters within a box enclosing the eastern boundary of the North Atlantic subtropical gyre. This estimate is based on the assumption that the box is in steady state, i.e., there is neither accumulation of organic carbon entering the box nor consumption of previously accumulated organic carbon. This assumption is acceptable for the budget of the central, intermediate, and deep waters as their decadal renewal times preclude seasonality. However, seasonal variability is pronounced in the surface layer and, hence, the steady state assumption could lead to biased values of the annual net fluxes. Furthermore, although the contribution of mesoscale phenomena needs to be addressed in future studies, our results show that a major fraction of the mesopelagic organic carbon demand in this area is fuelled by DOC. Indeed, the formation of MW has been found to be the main mechanism for the export of anthropogenic carbon in this region^38^. Despite the inaccuracy of the steady-state assumption the carbon budget presented here shows that the vertical entrainment of ENACW into subducting MOW contributes to the export of DOC to mesopelagic waters, where it may constitute the main substrate supporting dark ocean respiration. Likewise, due to the westward advection of intermediate waters, this process may also represent a significant supply of organic carbon to the eastern North Atlantic outside the box.

## Materials and Methods

### Hydrography and seawater sampling

Between October 15 and November 11, 2009 the RV *Hespérides* carried out an intensive hydrographic survey in the eastern boundary of the North Atlantic subtropical gyre at the confluence region between the Azores Current System, the Portugal Current and the Canary Current. A grid of 81 conductivity-temperature-depth (CTD) stations was distributed along a box region defined by 28.7–37.0°N and 24.5°W. At 47 of these stations seawater samples were taken for analysis of dissolved (DOC) and particulate organic carbon (POC; Fig. 1, numbered dots).

Readers interested in a detailed description of the hydrography and water mass characterization are referred to Pérez-Hernández and coworkers^44^.

### Organic carbon

Discrete samples of POC were obtained at selected depths from the surface up to the bottom (5, 25, 50, 100 200, 400, 600, 800, 1000, 1500, 2000, 3000, 4000 and 5000 m) by means of a rosette sampler equipped with twenty-four 10 L Niskin bottles. Although we are aware that particles sink at different rates, we assume that all the POC collected in oceanographic bottles corresponds to the slowly-settling or suspended pool (POC_susp_)^20^. Thus, this pool represents an upper threshold for the particulate organic material that is susceptible to lateral transport. Water samples (4 L) for POC_susp_ were collected in polypropylene bottles and filtered through precombusted (450 °C, 12 hours) 25 mm Whatman GF/F-filters (pore size 0.7 µm). The filters were wrapped in precombusted aluminium foil and frozen at −20 °C. In the laboratory, the filters were thawed and dried overnight at 55 °C, then placed overnight in a desiccator saturated with HCl fumes to remove inorganic carbon, and dried again in a second desiccator with silica gel for at least 24 hours. Finally, the filters were packed in tin sleeves before being analysed with a Perkin-Elmer 2400 CHN elemental analyser following standard protocols^94^. Unused pre-combusted GF/F-filters were treated in the same way and used as handling blanks. Blanks ranged from 0.06 to 0.7 µmol C L^−1^. DOC adsorption onto the filters was estimated at several random stations, at three different depth levels, as the amount of carbon retained on a backing filter placed underneath the main filter. The values ranged from 0.17 to 1.73 µmol C per 25 mm-diameter GF/F-filter, which are similar to previous estimates^20, 95, 96^.

At the same stations and depth levels, samples for the analyses of DOC were collected in 250 mL acid-cleaned all-glass flasks. Samples from the upper 100 m were immediately filtered through precombusted (450 °C, 4 hours) 47 mm Whatman GF/F filters in an acid-cleaned all-glass filtration system. Filtered surface and unfiltered deeper water samples were collected in 10 mL precombusted glass ampules (450 °C, 12 hours). After acidification with H_3_PO_4_, the ampoules were heat-sealed and preserved in the dark at 4 °C until analysis in the laboratory with a Shimadzu TOC-V organic carbon analyser by high temperature catalytic oxidation (HTCO). The system was calibrated daily with potassium hydrogen phthalate (99.95–100.05%, p.a., Merck). The precision of the DOC calibration was ± 1 μmol L^−1^. The performance of the instrument was tested with the carbon reference materials (CRM) provided by D. A. Hansell (University of Miami, USA). Measured concentrations of the CRM were 45.5 ± 1.7 µmol C (n = 10); the certified value is 44–46 µmol C L^−1^ (lot#09-06 from the Florida Strait at 700 m).

### Organic carbon budget

The water column was divided into discrete neutral density layers^97^. The upper four layers coincide roughly with the main thermocline waters (below 27.38 neutral density layers; 0–700 m), the following three layers with intermediate waters (27.38–27.92; 700–1500 m), and the lowest seven layers with deep waters (27.92–28.10; below 1500 m). Our estimates were based on the inverse box model used by Pérez-Hernández and coworkers^44^ to obtain mass transports (M) for each layer along the northern, western, and southern transects. The model uses the thermal wind equation after calculating the reference level velocities and their uncertainties. These velocities are calculated assuming geostrophy, and mass and property conservation. The inverse box model used has been extended from a previous one^98^ to include the approximate conservation of mass and anomalies of salinity and heat, and to allow transfer between layers. This model also considers adjustment of fresh water fluxes and Ekman transports in each section. The inverse problem consists of 37 equations and 130 unknowns: 99 for reference velocities, 26 for vertical velocities and vertical diffusion, 4 for Ekman transports and 1 for the freshwater flux. To solve the inverse problem, the Gauss-Markov method, which produces a minimum error variance solution from initial estimates of the unknowns, was used. M at each layer is multiplied by the averaged suspended and dissolved organic carbon concentrations (C) at the same layer to obtain the corresponding lateral carbon fluxes (M × C). To report these carbon fluxes, the layers were grouped into surface, central, intermediate, and deep waters. The lateral carbon fluxes were summed to obtain the corresponding carbon budgets for each layer.

The robustness of the carbon fluxes and budgets calculated in this study was tested by means of the following perturbation test (see supplementary material): carbon fluxes were calculated as (M ± erM) × (C ± erC), where erM and erC represent the uncertainty of the estimation of M and C, respectively. Therefore, the values of M and C are perturbed within the limits of the uncertainty of their respective estimations. A total of 100 perturbations were performed for each carbon flux and budget. The average of these 100 values is considered the optimum solution and the corresponding standard deviation (SD) an estimate of the uncertainty of the fluxes and budgets^99^. See Pérez-Hernández and coworkers^44^ for a thorough analysis of the values of M and erM used in the present calculations. Concerning the uncertainty of POC_susp_ and DOC measurements, these were obtained by calculating the SD of all suspended and dissolved organic carbon measured in each layer at each transect.

To obtain the organic carbon fluxes into/out of the Mediterranean Sea, mass transports across the Strait of Gibraltar were set at −0.78 ± 0.1 Sv for the surface outflow to the Mediterranean and 0.68 ± 0.1 Sv for the bottom inflow to the eastern North Atlantic^100^ (1 Sv = 10^9^ kg s^−^¹). These mass transports, which are within the range of other estimates in the literature^101–107^, were multiplied by the average concentrations of POC_susp_ (3.4 mmol m^−^³ in the outflow and 1.6 mmol m^−^³ in the inflow) and e-DOC (13.7 mmol m^−^³ in the outflow and 2.7 mmol m^−^³ in the inflow) measured in the same region by Arístegui and co-workers (unpublished data from May 2014).

Net respiration (R) of POC_susp_ and e-DOC within each layer of the box is obtained as follows:

1 $$\frac{\text{d}TOC}{\text{d}t}={\rm{I}}-{\rm{O}}-{\rm{R}}$$

Assuming steady state conditions (dTOC/dt = 0), the balance of inputs (I) minus outputs (O) of total organic carbon (TOC = POC_susp_ + e-DOC) across the open boundaries must equal the total net respiration within the box;, i.e., R = I − O.

### Data Availability

The datasets generated during and/or analysed during the current study are available from the corresponding author on reasonable request.

## Electronic supplementary material

Supplementary Information
