# Supplementary material for: Organic carbon budget for the eastern boundary of the North Atlantic subtropical gyre: major role of DOC in mesopelagic respiration
Source: Sci Rep. 2017 Aug 31;7:10129. doi: 10.1038/s41598-017-10974-y (PMC5578995; doi:10.1038/s41598-017-10974-y)
Supplement: Supplementary file 1 — Supplementary Information [file 41598_2017_10974_MOESM1_ESM.doc]

**Organic carbon budget for the eastern boundary of the North Atlantic subtropical gyre: major role of DOC in mesopelagic respiration**

Yeray Santana-Falcón, Xosé Antón Álvarez-Salgado, María Dolores Pérez-Hernández, Alonso Hernández-Guerra, Evan Mason, and Javier Arístegui
